# Alcohol Hangover Slightly Impairs Response Selection but not Response Inhibition

**DOI:** 10.3390/jcm8091317

**Published:** 2019-08-27

**Authors:** Antje Opitz, Jan Hubert, Christian Beste, Ann-Kathrin Stock

**Affiliations:** Cognitive Neurophysiology, Department of Child and Adolescent Psychiatry, Faculty of Medicine, TU Dresden, Fetscherstr. 74, 01307 Dresden, Germany

**Keywords:** alcohol, hangover, cognitive control, automatism, Simon Nogo task, response selection, response inhibition

## Abstract

Alcohol hangover commonly occurs after an episode of heavy drinking. It has previously been demonstrated that acute high-dose alcohol intoxication reduces cognitive control, while automatic processes remain comparatively unaffected. However, it has remained unclear whether alcohol hangover, as a consequence of binge drinking, modulates the interplay between cognitive control and automaticity in a comparable way. Therefore, the purpose of this study was to investigate the effects of alcohol hangover on controlled versus automatic response selection and inhibition. *N* = 34 healthy young men completed a Simon Nogo task, once sober and once hungover. Hangover symptoms were experimentally induced by a standardized administration of alcoholic drinks (with high congener content) on the night before the hangover appointment. We found no significant hangover effects, which suggests that alcohol hangover did not produce the same functional deficits as an acute high-dose intoxication. Yet still, add-on Bayesian analyses revealed that hangover slightly impaired response selection, but not response inhibition. This pattern of effects cannot be explained with the current knowledge on how ethanol and its metabolite acetaldehyde may modulate response selection and inhibition via the dopaminergic or GABAergic system.

## 1. Introduction

Alcohol hangover is an unpleasant state that may occur after an episode of heavy drinking, that is, once the breath/blood alcohol concentration (BAC) returns to 0.0‰. It subsumes several aversive mental and physical symptoms like headaches, vomiting, tiredness, sweating, circulatory problems or depressed mood [1]. It has furthermore been shown to slow psychomotor speed as well as information processing and has been suggested to impair several cognitive functions, including attention, memory, and executive functioning [2,3,4,5,6,7]. As a consequence, alcohol hangover is associated with impaired workplace productivity and safety [8], as well as reduced driving abilities [4,9]. Given that binge drinking is quite prevalent [10,11,12], alcohol hangover has been estimated to also be very prevalent and result in huge economic and societal costs due to its debilitating effects [2].

Importantly, regular binge-drinking does not only increase the likelihood and frequency of hangover, it also strongly increases the risk of developing alcohol use disorder (AUD) [13,14,15]. While it is still unclear whether or how these two consequences are functionally linked, it has been shown that the acute cognitive effects of a high-dose alcohol intoxication resemble the pattern of cognitive deficits observed in AUD patients—both produce pronounced impairments in cognitive control/executive functioning, while behavioural automaticity is comparatively preserved [16,17,18,19,20]. This imbalance between behavioural control and automaticity has repeatedly been shown to play an important role in the development and maintenance of AUD [21] but it has never been investigated whether such specific effects can also be found during alcohol hangover (which should be a regular occurrence in frequent binge drinkers). Demonstrating the persistence of such detrimental effects during hangover (i.e., beyond acute intoxication) would provide an important functional link, which may help to explain why and how regular binge drinking increases the risk of developing AUD [13,14,15]: If impairments of cognitive control functions persisted beyond acute intoxication, the poor cognitive functioning on the day following alcohol use might promote continued aberrant drinking as well as other decisions that may be detrimental to the overall health of affected individuals. This is especially relevant as acute alcohol intoxication, alcohol hangover and AUD are all characterized by changes in the dopaminergic and GABAergic neurotransmitter systems [22,23,24,25,26,27]. Specifically, ethanol and its major metabolite acetaldehyde, which has been suggested to strongly contribute to hangover symptoms [28,29], show similar effects on dopaminergic neural transmission by increasing dopaminergic signalling [23,26]. In contrast, ethanol enhances GABAergic signalling [24,25], while acetaldehyde has been suggested to decrease GABAergic signalling [27,30], even though this effect is still debated [31,32]. Overall, both the dopaminergic and GABAergic neurotransmitter systems play a strong modulatory role for cognitive control including response selection [33,34,35,36,37] and might therefore provide a functional link between all three phenomena.

We therefore set out to investigate the effects of alcohol hangover on controlled versus automatic behavior in young healthy males. They were subjected to a previously established, counter-balanced within-subject study design where hangover symptoms are experimentally induced via standardized administration of alcoholic beverages with high congener content [38]. In order to assess differences in controlled versus automatic response selection, we used the Simon Nogo task [39], which is an extended Simon task [40,41], where 30% of the trials require to inhibit all motor responses. The typical Simon effect reflects a stimulus-response (S-R) conflict [41,42]. This conflict is thought to arise between automatic “direct route” processes that promote “unconditionally automatic” responding on the task-irrelevant stimulus side and controlled “indirect route” processing of the correct response based on the “conditional” processing of task-relevant stimulus features [43]. When stimulus and responding hand have the same laterality (S-R congruency), these two kinds of processing indicate the same response. This overlap allows to rely on automatic response selection so that very little control is needed. When stimulus and responding hand have an incongruent laterality, the two processes interfere with each other. As a consequence, increased control is required to overcome the incorrect automatic response tendencies, which ultimately impairs response selection [40,41,43,44,45]. Aside from this typical Simon effect, opposing effects are observed in case of response inhibition which is typically better in case of S-R incongruence, than in case of S-R congruency [18,39,46]. The reason for this inversion of effects is that, other than response selection, response inhibition is typically worse in case of fast and automatic response tendencies (as compared to slower, more controlled responding) [18,39,47,48]. As a consequence, correct responding requires more top-down control/is more error-prone in incongruent Go trials and congruent Nogo trials (as compared to congruent Go trials and incongruent Nogo trials). Importantly, the detrimental effects of an acute high-dose alcohol intoxication have recently been found to be most pronounced in these conditions, suggesting that top-down control is more severely impaired by alcohol intoxication than automatic processing [18]. As we wanted to test whether the same effects can be found during alcohol hangover, we hypothesized that hangover induces a comparable pattern (i.e., stronger impairments in incongruent Go trials and congruent Nogo trials, as compared to congruent Go trials and incongruent Nogo trials). Given that alcohol hangover might however not necessarily replicate the same data pattern as acute intoxication [38], we decided to conduct Bayesian analyses on non-significant hangover effects in order to substantiate whether the null or alternative hypothesis is more likely, given the obtained data.

## 2. Experimental Section

### 2.1. Participants and Sample Size Estimation

In a previous within-subject hangover study conducted by our group [38], the detected hangover effects yielded effect sizes between $\eta_{p}^{2}$ = 0.26/*f* = 0.59 and $\eta_{p}^{2}$ = 0.39/*f* = 0.80. When using those, as well as the correlation of *r* = 0.356 among repeated (sober accuracy) measures obtained in a previous study using the same paradigm [18], for an a priori estimation of required sample size with G*power software [49], we obtained a sample size between *n* = 7 and *n* = 4, when yielding for an alpha error probability of 5% and a power of 95%. The effect sizes for acute high-dose intoxication on this task were between $\eta_{p}^{2}$ = 0.09/*f* = 0.31 and $\eta_{p}^{2}$ = 0.16/*f* = 0.44 [18], which yields samples sizes between *n* = 19 and *n* = 11 in the same a priori estimation. When being a little more conservative and assuming medium effect sizes of *f* = 0.25 [50], the a priori power analysis yielded a required sample size of *n* = 30. In order to compensate for drop-outs and minor issues, we therefore initially recruited *n* = 37 healthy male participants. These were aged 19–28 years and recruited via online advertising, flyers and postings at the local University (TU Dresden) and in the local nightlife district (Dresden Neustadt). All participants were right-handed and had normal or corrected to normal vision. Inclusion criteria and eligibility was assessed during a telephone screening. All included participants reported to have no chronic, somatic, neurological or psychiatric diseases and to not take any medication affecting normal central nervous system (CNS), liver or kidney function. We further assessed the alcohol use disorders identification test (AUDIT) [51]. In order to exclude individuals with a high likelihood of AUD and/or high alcohol tolerance, we excluded all applicants with an overall AUDIT score above 19 [51]. We further excluded all individuals reporting to binge-drink (i.e., consume 8 or more units of alcohol on a single occasion), or have alcohol-induced memory issues, or fail to do things that were normally expected from them “daily or almost daily.” In order to exclude light drinkers (who might not be able to cope well with the alcohol amounts administered in this study), we excluded individuals who indicated to binge-drink only once a month or less in the last 12 months. As a consequence, the minimum AUDIT cutoff for inclusion was 2 points. Lastly, individuals who stated to not having been noticeably drunk on at least one occasion in the last 12 months were also excluded. All participants provided written informed consent and were reimbursed with 80 €. The study was approved by the Ethics Committee of the Faculty of Medicine of the TU Dresden and was conducted in accordance of the Declaration of Helsinki.

### 2.2. Experimental Design and Hangover Provoking Procedure

**Figure 1:**
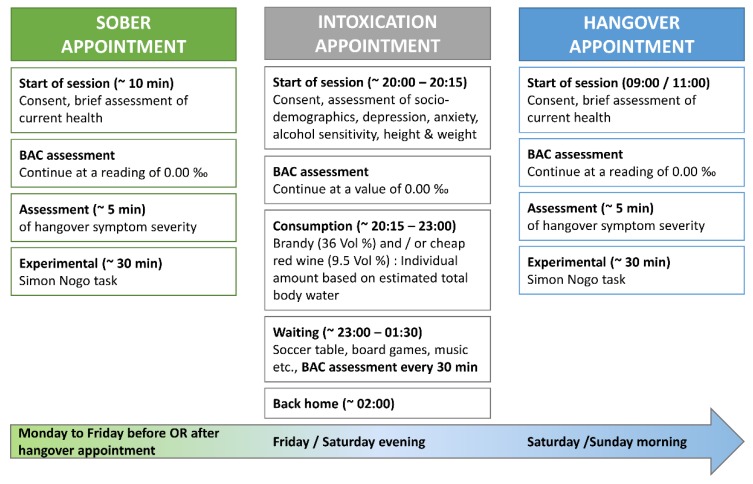
Illustration of sober, intoxication and hangover appointments, as adapted from Zink et al. [38]. Each participant was tested on a sober and a hangover appointment, which were at least 48 h and no more than 7 days apart. The order of both appointments was counterbalanced across the sample. Each session started with providing written consent and a brief assessment of the current health. Data collection (assessment of hangover symptoms and paradigm performance) was not started before participants had reached a blood alcohol concentration (BAC) of 0.00‰ on both appointments. In order to provoke alcohol hangover symptoms, participants were invited to the laboratory on a Friday or Saturday evening at 20:00. After providing their written consent, participants filled out questionnaires assessing sociodemographic data, depression, anxiety, alcohol sensitivity, height and weight. To assure sobriety at the start of drinking, BAC was assessed before alcohol consumption. An individually calculated amount of alcohol was consumed from around 20:15 to 23:00. Participants were asked to stay in the laboratory until 01:30. BAC readings were taken every 30 min starting half an hour after the last sip of alcohol. Eventually, participants were taken home by taxi and returned the next morning between 09:00 and 11:00 for their hangover appointment.

The study design and experimental intoxication to provoke hangover symptoms followed the protocol used in a previous study of our lab [38], which is illustrated in Figure 1.

In short, each participant was tested twice (once sober and once hungover) with a delay of no less than 48 h and no more than 7 days between sober and hangover appointment. The order of both appointments was counterbalanced between all participants (that is, half of the participants had their sober appointment before their hangover appointment; the other half had their hangover appointment before their sober appointment). Breath alcohol concentration (BAC) was assessed at the start of each appointment. The experiment was not started until participants reached a BAC of 0.00‰. We used the breathalyser “Alcotest 3000“ to measure BAC as instructed by the manufacturer (Drägerwerk, Lübeck, Germany). In order to provoke hangover symptoms, we invited 4 to 8 participants to our laboratory on the night before their hangover appointment. These drinking appointments were always scheduled for Friday or Saturday evening (20:00 starting time; ending usually 01:30 to 02:00), while the subsequent hangover appointment was always scheduled for the morning of the following day (i.e., either Saturday or Sunday, starting time between 09:00 and 11:00). This resulted in a slight reduction of sleeping time, which seems to be inversely associated with hangover severity [52].

In the night of experimental intoxication, an individual amount of alcohol was determined for each participant using a version of the equation by Widmark [53] and Watson [54]. We aimed to reach BAC values of no more than 1.6‰, by assessing how much alcohol needed to be added to the total body water in order to reach a concentration of 2.0‰. Given that the expected resorption deficit is about 20% on an empty stomach in case all alcohol is consumed at once, this ensures that a BAC of 1.6‰ is very unlikely to be exceeded. Furthermore, the consumption duration was stretched over at least 2 h by the experimenters and all participants were instructed to partake on a full stomach, where the resorptions deficit is usually higher (about 30–40%). Hence, participants were expected to reach a mean BAC of approximately 1.2‰ with a small probability of achieving a BAC beyond 1.6‰. The tool used to determine individual amounts of alcohol and to document alcohol consumption can be found online at https://osf.io/9ykpg/.

Due to the greater likelihood of causing a severe hangover, only alcoholic drinks with a high congener content were offered [55,56]. Therefore, each participant could choose between drinking cheap brandy (36 Vol %) and/or cheap red wine (9.5 Vol %). Both drinks were served by the experimenters in standardized portions of 200 mL red wine (15 g alcohol) or 50 mL brandy (14 g alcohol), so that the speed and amount of alcohol consumption were similar across drinks. Participants could furthermore choose whether they wanted to consume each drink pure, chilled on ice or mixed with caffeine-free coke, ginger ale, or orange lemonade. Tap water and snacks (chips, wine gums) were available at all times and their intake was not controlled or documented. Moreover, participants were permitted to smoke while drinking, because this is assumed to increase hangover symptoms [57,58]. This opportunity was taken by *n* = 8 participants, of which *n* = 7 claimed to be regular smokers. BAC was measured 30, 60, 90 and 120 min after the end of alcohol consumption. Lastly, participants were encouraged to neither use caffeine, guarana nor nicotine within four hours before the start of each appointment.

### 2.3. Questionnaires

At the beginning of the intoxication appointment (before alcohol administration), participants provided sociodemographic details and filled in the anxiety sensitivity index (ASI) [59] to assess fears of physical symptoms of anxiety itself. As hangover symptoms like nausea, heart racing, shivering or concentration problems are similar to the anxiety symptoms assessed by the ASI, this allowed us to obtain an estimation of how unpleasant physical symptoms of hangover might be for the participant (please note that we did so as there is currently no reliable questionnaire to assess the affective rating of hangover symptoms). Moreover, Beck’s depression inventory (BDI) [60] was used to determine depressive symptoms, as these may have a potential influence on cognitive (task) performance. To evaluate sensitivity to common alcohol effects, participants were asked to fill in the alcohol sensitivity questionnaire (ASQ) [61]. The ASQ enables to distinguish between alcohol related experiences referring to lighter drinking (like being more talkative) and alcohol related experiences referring to heavier drinking (like experiencing a hangover). High ASQ scores point to low alcohol sensitivity. At the beginning of each sober and hangover appointment, participants were asked to rate their hangover symptom severity on a Likert-scale ranging from 0 (no symptoms) to 10 (extreme symptoms) consisting of 23 items as used by van Schrojenstein Lantman and colleagues [1]. Of note, we added another item to assess sleeping problems/sleep quality. Finally, participants stated how many hours of sleep they obtained the previous nights, respectively.

### 2.4. Task

**Figure 2:**
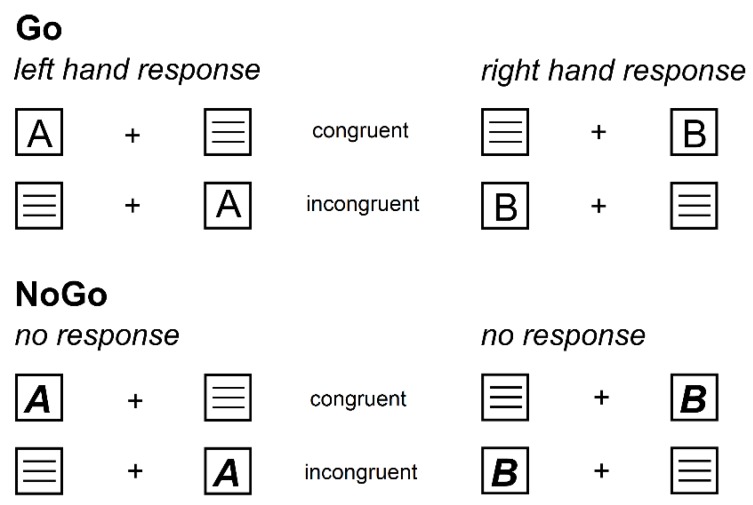
Illustration of the experimental paradigm showing all possible stimulus combinations. On the upper half, the Go condition (70% of all trials) is illustrated. It was indicated by regular letter stimuli. Regardless of stimulus location, stimulus “A” always required a left button press while stimulus “B” always required a right button press. Whenever stimulus location and required response button were located on the same side, the trial was labelled as congruent. Whenever stimulus location and required response button were located on opposite sides, the trial was labelled as incongruent. On the lower half, the Nogo condition (30% of all trials) is illustrated. It was indicated by bold and italic letter stimuli (“***A***” and “***B***”), which required to withhold all motor responses and thus display no response at all.

We used a so-called Simon Nogo paradigm in order to assess the effects of alcohol hangover on automatic versus top-down response selection and inhibition [39]. Importantly, we previously employed this paradigm in a study demonstrating that an acute alcohol intoxication of ~1.1‰ had stronger detrimental effects when high levels of top-down control were required for correct responding, as compared to less controlled/more automatic response processing [18]. The paradigm is schematically illustrated in Figure 2.

The task was presented on a 17’’ CRT monitor, which displayed a central white fixation cross and two lateralized white frame boxes on black background throughout the entire duration of the task. Every trial started with the synchronous 200 ms presentation of a target stimulus (single yellow letter; either “A” or “B”) in one of the two boxes and a distractor stimulus (three white horizontal lines) in the respective other box. Participants were instructed to press the left Ctrl button on a regular QWERTZ keyboard with the left index finger in response to the target letter “A” and to press the right Ctrl button with the right index finger in response to the target letter “B.” Importantly, participants were further instructed to only respond when the target letters were printed in a regular font (i.e., “A”/“B”; Go condition) but to refrain from all responses whenever the target letters were printed italic and bold (i.e., “***A***”/“***B***”; Nogo condition). The first given response ended the trial. In Nogo trials, any response was coded as a false alarm, while it was either coded as “correct” or “incorrect” in Go trials. If no response was given, the trial was terminated after 1700 ms and either coded as a “miss” (Go condition) or as a “correct omission” (Nogo condition). The inter-trial interval (ITI) was jittered between 1300 and 1700 ms. Overall, the experiment comprised 360 trials, which were subdivided into three equally large blocks. The participants were offered breaks in between the blocks and took approximately 15 min to complete the task.

Of note, each of the two target stimuli occurred equally often on each side and in each condition. Whenever the target stimulus appeared on the side of the correct response (i.e., left “A” and right “B”), we coded the trial as congruent (50% of Go and Nogo trials). When the target stimulus appeared on the opposing side (i.e., right “A” and left “B”), we coded the trial as incongruent (50% of Go and Nogo trials). In summary, each experimental block contained 70% Go trials and 30% Nogo trials and in each of those two conditions, each target letter and congruency rating occurred equally often. The order of trials was separately randomized in each block.

### 2.5. Statistical Analyses

The behavioural data (accuracy) was analysed using a repeated-measures ANOVA. Hangover status (hangover vs. sober), condition (Go vs. Nogo), and congruency (congruent vs. incongruent) were used as within-subject factors. Reported values of the ANOVA underwent Greenhouse-Geisser correction and parametric post hoc tests were Bonferroni-corrected, whenever necessary. All descriptive statistics were reported using the mean value and the standard error of the mean (SEM) as a measurement of variability. All variables were tested for normal distribution using Kolmogorov-Smirnov tests. As this prerequisite was not given for most accuracy variables, all significant main effects and post hoc tests were additionally tested for significance using non-parametric Wilcoxon signed-rank tests. Lastly, in order to investigate whether the null or alternative hypothesis was more likely in case of non-significant main or interaction effects of hangover status, Bayesian analyses were computed as suggested by Masson [62]. In short, this method returns the likelihood of the H_0_ and H_1_, given the obtained data. According to Raftery [63], P(H_i_|D) values of 50–75% can be regarded as weak evidence for a given hypothesis, values of 75–95% can be regarded as positive evidence for a given hypothesis, values of 95–99% can be regarded as strong evidence for a given hypothesis, and values above 99% can be regarded as very strong evidence for a given hypothesis. Behavioural data and all statistical analyses can be found online at https://osf.io/9ykpg/.

## 3. Results

### 3.1. Sample Characteristics

Out of the initially recruited *n* = 37 participants, one participant was excluded from the sample due to a high residual BAC at the start of his hangover appointment (0.45‰), as this would have required 4 to 5 h of waiting time which he could not afford to spend on that day. Another participant was excluded due to technical problems on the sober appointment. A last participant was excluded due to performance accuracy below 60% in Nogo trials on both appointments (his performance deviated from the mean performance of the respective conditions by ≥ 3.57 standard deviations). As a result, *n* = 34 participants entered statistical analyses. Of those, *n* = 17 had their sober appointment before their hangover appointment and *n* = 17 had their hangover appointment before their sober appointment. Sociodemographic characteristics as well as hangover-related data are provided in Table 1. Subjective ratings of sleep and hangover symptoms on both appointments are given in Table 2.

### 3.2. Test-Retest Reliability of the Experimental Paradigm

As we tested the participants on two consecutive appointments, we decided to assess the test-retest reliability of the Simon Nogo paradigm. For this, we compared the first and second appointment of the entire group, while disregarding the experimental manipulation. As appointment order had been balanced across the group, half of the performance data in the first appointment was “sober,” while the other half was “hungover.” The same was true for the second appointment. We separately determined the test-retest reliability of Go and Nogo trials, as they assess different cognitive domains. Doing so, we found that the test-retest reliability was good in both measures, as all *r* ≥ 0.803 and all *p* < 0.001.

### 3.3. Behavioral Data

**Figure 3:**
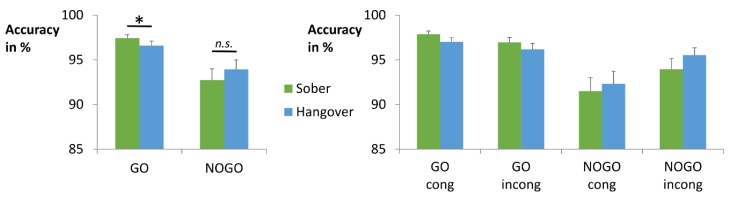
Both graphs visualize the obtained sober vs. hungover accuracy (percentage of correct trials) with error bars depicting the standard error of the mean. Left graph: While we obtained no significant difference between the sober and hangover appointments, Bayesian statistics strongly suggested that there was an interaction between hangover status and condition. As depicted, post hoc paired *t*-tests detailing this effect showed significantly decreased accuracy in the Go condition, but not in the Nogo condition. Right graph: All factor combinations are depicted. There was a significant interaction between condition and congruency, as Go accuracy was higher in the congruent condition than in the incongruent condition, while Nogo accuracy was higher in the incongruent condition than in the congruent condition. This effect did, however, not vary between sober and hangover appointments.

The accuracy data are shown in Figure 3. The repeated-measures ANOVA for accuracy revealed a main effect of condition (*F*_(1,33)_ = 12.284; *p* = 0.001; $\eta_{p}^{2}$ = 0.271), with higher accuracy in Go (97.0% ± 0.40) than in Nogo trials (93.3% ± 1.03). There was also a main effect of congruency (*F*_(1,33)_ = 6.944; *p* = 0.013; $\eta_{p}^{2}$ = 0.174), showing higher accuracy in incongruent (95.7% ± 0.54) than in congruent trials (94.7% ± 0.68). Yet, it should be noted that this effect does not contradict the Simon effect, as these numbers average Go trials (where congruent trials are typically performed better than incongruent trials) and Nogo trials (where congruent trials are typically performed worse than incongruent trials). In addition, there was an interaction of condition x congruency (*F*_(1,33)_ = 16.03; *p* < 0.001; $\eta_{p}^{2}$ = 0.327). In line with previous studies of this task [18,39], we found opposing effects of stimulus congruency on Go versus Nogo trials. Specifically, there was a typical Simon effect (congruent > incongruent) in Go trials (*t*_(33)_ = 2.134; *p* = 0.040; cong = 97.4% ± 0.33; incong = 96.6% ± 0.54), which was inverted (congruent < incongruent) in Nogo trials (*t*_(33)_ = −3.851; *p* = 0.001; cong = 91.9% ± 1.30; incong = 94.7% ± 0.84). In line with this, a post-hoc paired *t*-test showed a positive Simon effect (congruent minus incongruent) in Go trials (0.9% ± 0.40) and negative Simon effect in Nogo trials (−2.8% ± 0.74), which significantly differed from each other (*t*_(33)_ = 4.004; *p* < 0.001). The interaction of hangover status x condition was non-significant (*F*_(1,33)_ = 3.478; *p* = 0.071; $\eta_{p}^{2}$ = 0.095) but add-on Bayesian analysis provided positive evidence for the alternative hypothesis, given the obtained data (P_BIC_(H_1_|D) = 94.3%) (please see Table 3 for details) [63]. We therefore decided to conduct post-hoc analyses. Post-hoc paired *t*-tests showed significantly higher accuracy in sober Go trials (97.4% ± 0.39) than in hangover Go trials (96.6% ± 0.51) (*t*_(33)_ = 2.053; *p* = 0.048). No such effect was obtained for Nogo trials (*t*_(33)_ = −1.125; *p* = 0.269). Additional correlation analyses showed that hangover Go accuracy was significantly correlated with the hangover effect (sober minus hangover) (*r* = −0.664, *p* < 0.001), while sober Go accuracy was not (*p* = 0.291). This suggests that the size of the hangover effect was mainly determined by changes induced by the hangover status. In order to explore whether these measures showed a performance difference between participants who reported light versus heavy hangover symptoms, we performed a median split of the group. All participants with an overall hangover severity rating of 0–3 on the 11-point Likert scale were classified as “low hangover severity” (*n* = 19), while all participants with an overall hangover severity rating of 4–10 were classified as “high hangover severity” (*n* = 15). Mann-Whitney-U tests for independent samples showed that Go accuracy on the hangover day was lower in individuals with high hangover severity (95.50% ± 0.89) than in individuals with low hangover severity (97.43% ± 0.51) (*p* = 0.040). No such effect was found for the Go accuracy performance difference between sober and hangover day (*p* = 0.891). All other main and interaction effects of the hangover status were non-significant in the repeated-measures ANOVA (all *F* ≤ 0.364; *p* ≥ 0.550).

In order to assess whether there was truly no other effect of hangover status (i.e., whether the null hypothesis H_0_ was more likely than the alternative hypothesis H_1_), we furthermore ran add-on Bayesian analyses [62] for all main and interaction effects of the hangover status. With the exception of hangover status x condition (on which we already elaborated above), all Bayesian analyses provided positive evidence for the null hypothesis, that is the assumption that hangover status did indeed not modulate the data (please see Table 3 for details).

In summary, we found no significant hangover effects on task performance. Yet, Bayesian analyses suggested a slight detrimental effect of hangover status on response selection, as Go accuracy was slightly worse at the hangover appointment than at the sober appointment. This effect was slightly larger for individuals with high hangover severity on the day of hangover testing. Given that no such effect was observed for the Nogo condition, there was no such corresponding effect on response inhibition.

### 3.4. Add-On Analyses—Alcohol Sensitivity

Previous studies suggested that individuals with lower sensitivity to the effects of alcohol are more hangover-resistant [64] and might show altered cognitive control processes in alcohol-related contexts [65,66]. It could hence be possible that alcohol sensitivity modulated hangover effects in our study. Based on this, we had a closer look at alcohol sensitivity in add-on analyses, as described below.

In total, *n* = 2 participants stated in the ASQ that they had never experienced a hangover before (first item). Yet, only one of these two participants reported not having overall hangover symptoms on the day of the hangover appointment (rating of “0” on the hangover severity scale by van Schrojenstein Lantman et al. [1]). Despite this, he still indicated mild complaints in some of the symptoms that were separately assessed in the list by van Schrojenstein Lantman et al. [1]. Further given that none of the participants rated all of the assessed hangover symptoms as “0” on the hangover day, we concluded that none of them were truly hangover-resistant, as defined by never experiencing any kind of hangover symptoms on the day after heavy drinking. Lastly, we correlated the total ASQ score, the ASQ heavy score and the ASQ hangover item (maximal number of drinks which could be consumed before experiencing hangover) with the overall hangover severity Likert scale rating on the hangover test day. This showed no significant correlations (all uncorrected *p* ≥ 0.150).

In order to compare the self-reported number of drinks in the ASQ hangover item with the individually calculated amount of brandy (36 Vol %) administered during the intoxication appointment, we converted the latter into standard drink units (25 mL brandy or 100 mL red wine, which equal 7–7.5 gr of alcohol, were defined as one standard drink). A paired *t*-test revealed that the administered amount of alcohol exceeded the self-reported hangover threshold, as the number of experimentally administered standard drinks (16.81 drinks ± 0.25) was significantly larger than the maximal number of drinks which could be consumed before developing a hangover (9.09 drinks ± 0.69) (*t*_(31)_ = −10.388; *p* < 0.001).

In order to investigate whether alcohol sensitivity or hangover sensitivity correlated with the achieved intoxication, we correlated the overall ASQ score, the ASQ heavy score and ASQ hangover item with the BAC measured 30/60/90/120 min after the end of consumption on the evening of experimental drinking. None of the uncorrected correlations between ASQ and BAC were significant (all *p* ≥ 0.192).

Lastly, we investigated whether alcohol sensitivity or hangover sensitivity correlated with the relevant behavioural parameters. For this, we correlated the overall ASQ score, the ASQ heavy score and ASQ hangover item with the Go accuracy at the sober appointment, the Go accuracy at the hangover appointment and the Go accuracy difference between both appointments. None of the uncorrected correlations between ASQ and performance were significant (all *p* ≥ 0.068).

In summary, we found that none of the included participants were entirely resistant to hangover symptoms after high-dose alcohol administration. We further found that alcohol and hangover sensitivity did not significantly correlate with intoxication levels, subjective hangover ratings or performance in hangover-affected cognitive domains. Yet, it should be noted that the lack of such effects could potentially have been caused by the standardized alcohol administration (i.e., participants were not allowed to drink ad libidum) and/or the extremely homogenous study sample.

## 4. Discussion

Alcohol hangover is an unpleasant state that is reached after an episode of heavy drinking and starts once breath and blood alcohol levels have returned to zero [1]. Aside from symptoms like headaches and gastrointestinal problems [1], a wide range of mild cognitive impairments have been reported, including attention, memory, task-switching, and psychomotor performance deficits [2,3,4,5,7,67,68]. Importantly, these domains are also known to be impaired during acute alcohol intoxication [11,17,38,69,70]. It has furthermore been shown that both intoxication and hangover are at least partly characterized by similar neurobiochemical changes, like increased dopaminergic signalling [23,26]. Against this background, cognitive functions that are impaired by acute intoxication might also be impaired during alcohol hangover. Yet, the reports on this are still somewhat inconsistent, with some studies finding comparable effects, while others fail to do so [6,11,20,38,67,71,72]. Given that alcohol has repeatedly been reported to impair controlled behaviour to a larger degree than automatic behaviour [21], we decided to focus on how alcohol hangover impairs controlled versus automatic response selection processes. We experimentally induced a standardized intoxication and investigated whether the subsequent alcohol hangover impaired controlled response selection more severely than automatic response selection, as previously reported for acute alcohol intoxication using a Simon Nogo task [18]. Participants were tested once sober and once hungover (always at a BAC of 0.00‰).

Replicating the typical task effect of the Simon Nogo paradigm and thus further underpinning its validity, we found inverse effects of S-R congruency on response selection and response inhibition during both appointments [18,39,73]: Whenever automatic S-R mapping yielded the correct response, it was beneficial for response selection, but detrimental for response inhibition. Whenever automatic S-R mapping yielded the wrong response, the compensatory controlled S-R mapping was detrimental for response selection, but beneficial for response inhibition. These findings are in line with the assumptions of the dual-process theory [43]—In the context of lateralized stimuli, it distinguishes between “direct route” processes and “indirect route” processes. The theory postulates that the task-irrelevant stimulus lateralization gives rise to direct route processes that result in rather automatic response tendencies on the side of the stimulus. In contrast to that, indirect route processing refers to the controlled selection of the correct response based on task-relevant stimulus features (in our case letter identification) [43]. As direct route processing is faster and more expedient than indirect route processing, it improves response selection whenever there is an overlap between the two processes (i.e., in congruent trials, where both indicate the same response). Hence, response selection is improved by mostly relying on direct route processes in case of congruent stimuli. In case of incongruent stimuli, responses are typically slower and more error-prone as indirect route processing is required to overcome the wrong automatic response tendency. In contrast to response selection (Go trials), response inhibition (Nogo trials) is typically most impaired by fast and automatic response tendencies [18,39,47,48]. As a consequence, inhibition worsens whenever response selection is mainly driven by direct route processing (i.e., in case of congruent trials). In case of stimulus incongruence, the more controlled indirect route processing reduces automatic response tendencies, which supports response inhibition and ultimately improves performance.

In this context, the adverse effects of acute high-dose alcohol intoxication on response selection and inhibition have recently been shown to be most pronounced when high levels of control and effort were needed for correct task performance (i.e., in incongruent Go trials and in congruent Nogo trials) [18]. This suggests that top-down control is more strongly impaired by alcohol intoxication than automatic response selection processing. In terms of our main research question, we expected alcohol hangover to induce a comparable pattern of adverse effects. However, our data showed that the interaction between condition and congruency was not significantly modulated by hangover status. Furthermore, add-on Bayesian analysis provided positive evidence for rejecting the assumption that alcohol hangover alters the interplay between controlled and automatic processes in a similar fashion as intoxication. As both acute alcohol intoxication and AUD show similar impairment patterns (i.e., strongly impaired control functions and relatively preserved behavioural automaticity) [16,17,18,19,20], we had initially hypothesized that alcohol hangover might be characterized by similar changes, thus providing a functional link that helps to explain why and how regular binge drinking increases the risk of developing AUD [13,14,15]. Specifically, we had reasoned that cognitive control impairments which persist beyond intoxication, might contribute to poor behavioural choices, thus (partly) explaining the increased AUD risk of regular binge-drinkers. Given that we obtained positive evidence for the absence of such effects for congruency on its own, as well as the interaction of congruency and condition, the hypothesis of comparable effects between intoxication and hangover needs to be rejected. With respect to our initial hypothesis, this suggests that premorbid deficits in cognitive control, especially in the domain of inhibition, might contribute more to the observed link between binge-drinking and AUD, than the effects of repeated alcohol hangover. While our data does not provide concise information on why we did not find this effect, it seems conceivable that our participants were either not impaired, or healthy enough to compensate detrimental effects of hangover onto cognition (e.g., by increasing their effort during task performance).

Even though top-down cognitive control does hence not seem to be impaired by alcohol hangover, we found evidence suggesting that response selection might be slightly impaired. While we only observed a non-significant trend for this effect, Bayesian analyses strongly suggested that alcohol hangover seems to impair response selection in Go trials, but not response inhibition in Nogo trials. Importantly, this was observed regardless of S-R mapping. While these findings are not in line with our initial hypotheses, they match previous reports that alcohol hangover reduces information processing efficiency during response selection and increases the threshold for response execution in a number categorization task [5]. Importantly, the observed detrimental effect of hangover on response selection was slightly larger in individuals with high hangover severity, as compared to individuals with low hangover severity. Taken together, these findings might indicate a compensatory strategy for perceived deficits during hangover, where participants are more cautious and hesitant to respond. This also matches our descriptive data (please see Figure 3): Although we did not find significant hangover effects in the Nogo condition, alcohol hangover appears to have slightly improved response inhibition, rather than impairing it. The neurobiological basis of this, however, remains rather unclear, as we did not directly measure correlates of neurotransmission or neurotransmitter levels. It has been suggested that acetaldehyde, which is likely present during alcohol hangover, increases dopaminergic signalling but decreases GABAergic signalling [27]. But as dopamine and GABA have beneficial effects on both response selection and response inhibition [35,36,37,74,75], this does however not help to explain the finding of slightly impaired selection and relatively preserved inhibition.

There are two limitations pertaining to sex and age. We refrained from recruiting females as the ethical approval for this was denied. On average, females tend to have a slower metabolism of alcohol, which might lead to greater overall impairments [11]. Furthermore, heavy drinking has been shown to impair response inhibition more strongly in females than in males [76]. For these reasons, females could be likely to show greater deficits in cognitive performance during alcohol hangover. We also did not recruit adults over the age of 30 in order to have a homogeneous young and healthy study sample. Alcohol hangover frequency and the quality of hangover symptoms seem to change with age [77,78]. Hence, further studies considering both sexes as well as middle-aged to older adults are needed to investigate whether the hangover effects we observed may be generalized. Furthermore, we did not explicitly record participants’ history of binge drinking beyond the last 12 months, which were assessed with the AUDIT. We also did not assess details on hangover history beyond the ASQ. Assessing this data in more detail might have helped to explain differences in hangover severity and cognitive performance during hangover. As we pre-selected individuals according to their drinking habits, we however had a very homogenous sample, which likely showed only minimal variation with respect to this factor. Lastly, we omitted placebo administration, as it would have been rather easy to distinguish between placebo and alcoholic drinks, given the large amounts we served. In line with this, a recent study demonstrated no expectancy effects on cognitive performance during alcohol hangover by briefing one group of participants with the correct study purpose [79].

## 5. Conclusions

In summary, we investigated whether and how alcohol hangover modulates controlled versus automatic response selection and behaviour. For this, we used a Simon Nogo task that has previously been used to demonstrate stronger intoxication-related impairments in controlled behaviour than in automatic behaviour. The lack of significant hangover effects shows that alcohol hangover did not produce the same functional impairments as an acute high-dose intoxication. Yet, add-on Bayesian analyses suggested that alcohol hangover slightly impairs response selection but not response inhibition. These analyses also showed that alcohol hangover does most likely not alter controlled versus automatic response selection processes. As the pattern of effects is thus not comparable with intoxication or AUD, cognitive alcohol hangover symptoms cannot help to explain why or how regular binge drinking increases the risk of developing AUD.

## Figures and Tables

**Table 1:** Sociodemographic and alcohol-related data of all included participants. All values are given as means ± standard error of the mean (range). Of note, missing alcohol sensitivity questionnaire (ASQ) values (in case a participant indicated never having experienced a given alcohol-associated phenomenon) were not interpolated. All ASQ lighter drinking items as well as almost all ASQ heavier drinking items except for the item “passing out” were averaged, as only one participant reported ever having passed out after heavy drinking.

Characteristic	Included Sample (*n* = 34)
Age in years	23.21 ± 0.48 (19–28)
Height in cm	181.59 ± 0.99 (170–195)
Weight in kg	77.37 ± 1.73 (63–105)
Cigarettes smoked per day	0.80 ± 0.38 (0–10)
Hours of sport per week	4.65 ± 0.58 (0–16)
BDI Score	3.32 ± 0.70 (0–19)
ASI Score	13.34 ± 1.45 (1–33)
AUDIT Score	9.94 ± 0.54 (5–18)
ASQ Score total	8.40 ± 0.41 (3.25–13.29)
ASQ Score of light-drinking	5.27 ± 0.31 (1.63–9.25)
ASQ Score of heavy-drinking	13.43 ± 0.79 (5.20–24.00)
Individual measured alcohol amount of brandy (36 Vol %) in mL	419.71 ± 5.95 (369–516)
Alcohol consumption duration in minutes	182.50 ± 4.33 (111–243)
BAC 30 min after end of consumption	1.31 ± 0.03 (1.05–1.69)
BAC 60 min after end of consumption	1.24 ± 0.02 (1.01–1.56)
BAC 90 min after end of consumption	1.15 ± 0.02 (0.91–1.40)
BAC 120 min after end of consumption	1.07 ± 0.03 (0.83–1.43)

BDI = Beck Depression Inventory, ASI = Anxiety Sensitivity Index, AUDIT = Alcohol Use Disorders Identification Test, ASQ = Alcohol Sensitivity Questionnaire, BAC = Breath Alcohol Concentration.

**Table 2:** Subjective sleep and hangover symptoms on both appointments. Hangover symptoms were rated on an 11-point Likert scale ranging from 0 (no symptoms) to 10 (extreme symptoms). Of note, participants were asked to truthfully rate the severity of each symptom on both testing days, irrespective of whether they had been drinking the night before the sober appointment. The mild symptom severity for the sober appointment and the resulting minimal variance of this rating may have contributed to the fact that nearly all hangover symptoms differed significantly between the sober and the hangover testing (as was intended by the study). These comparisons were run with uncorrected paired *t*-tests. *p*-values are given in the column “Difference.” All values are reported as means ± standard error of the mean (range).

Symptom	Sober	Hangover	Difference
Hours of sleep in previous night	7.35 ± 0.15 (5.50–9)	5.58 ± 0.19 (4–8)	*p* < 0.001 **
Overall hangover severity	0 ± 0 (0–0)	3.82 ± 0.42 (0–10)	*p* < 0.001 **
Headache	0.06 ± 0.04 (0–1)	2.68 ± 0.40 (0–8)	*p* < 0.001 **
Nausea	0.03 ± 0.03 (0–1)	1.71 ± 0.39 (0–7)	*p* < 0.001 **
Concentration problems	0.61 ± 0.18 (0–4)	3.21 ± 0.40 (0–8)	*p* < 0.001 **
Regret	0.12 ± 0.12 (0–4)	1.00 ± 0.36 (0–10)	*p* = 0.003 **
Sleepiness	0.97 ± 0.22 (0–4)	3.91 ± 0.44 (0–9)	*p* < 0.001 **
Heart pounding	0.30 ± 0.10 (0–2)	0.94 ± 0.24 (0–5)	*p* = 0.010 *
Vomiting	0.03 ± 0.03 (0–1)	0.82 ± 0.34 (0–9)	*p* < 0.030 *
Tired	1.09 ± 0.21 (0–4)	4.71 ± 0.40 (1–10)	*p* < 0.001 **
Shivering	0.36 ± 0.13 (0–3)	1.24 ± 0.29 (0–6)	*p* = 0.006 **
Clumsy	0.42 ± 0.15 (0–3)	2.12 ± 0.33 (0–6)	*p* < 0.001 **
Weakness	0.30 ± 0.11 (0–2)	2.50 ± 0.37 (0–10)	*p* < 0.001 **
Dizziness	0.03 ± 0.03 (0–1)	1.88 ± 0.33 (0–8)	*p* < 0.001 **
Apathy	0 ± 0 (0–0)	1.03 ± 0.23 (0–5)	*p* < 0.001 **
Sweating	1.06 ± 0.28 (0–6)	1.24 ± 0.32 (0–9)	*p* = 0.555
Stomach pain	0.24 ± 0.16 (0–5)	1.29 ± 0.38 (0–8)	*p* < 0.001 **
Confusion	0.15 ± 0.08 (0–2)	1.15 ± 0.28 (0–7)	*p* = 0.001 **
Sensitivity to light	0.30 ± 0.13 (0–3)	1.68 ± 0.33 (0–8)	*p* < 0.001 **
Thirst	0.79 ± 0.22 (0–4)	3.44 ± 0.37 (0–8)	*p* < 0.001 **
Heart racing	0.15 ± 0.08 (0–2)	0.65 ± 0.26 (0–6)	*p* = 0.084
Anxiety	0.48 ± 0.16 (0–3)	1.24 ± 0.35 (0–9)	*p* = 0.016 *
Depression	0.21 ± 0.16 (0–5)	0.76 ± 0.29 (0–9)	*p* = 0.011 *
Reduced appetite	0.21 ± 0.16 (0–5)	2.12 ± 0.44 (0–9)	*p* < 0.001 **
Sleeping problems	0.21 ± 0.14 (0–4)	1.00 ± 0.35 (0–8)	*p* = 0.018 *

* *p* < 0.05, ** *p* < 0.01.

**Table 3:** Bayesian analyses for each effect of hangover status.

Effect	BF	P_BIC_(H_0_|D)	P_BIC_(H_1_|D)
Main effect hangover status	5.56	0.848	0.152
Interaction hangover status x condition	0.06	0.057	0.943
Interaction hangover status x congruency	5.77	0.852	0.148
Interaction hangover status x condition x congruency	5.92	0.855	0.145

BF = Bayes factor, P_BIC_(H_0_|D) = posterior probability for the null hypothesis (i.e., the probability of the null hypothesis, given the obtained data); P_BIC_(H_1_|D) = posterior probability for the alternative hypothesis.
